# Mesaconase/Fumarase FumD in *Escherichia coli* O157:H7 and Promiscuity of *Escherichia coli* Class I Fumarases FumA and FumB

**DOI:** 10.1371/journal.pone.0145098

**Published:** 2015-12-14

**Authors:** Miriam Kronen, Ivan A. Berg

**Affiliations:** Mikrobiologie, Fakultät für Biologie, Albert-Ludwigs-Universität Freiburg, Freiburg, Germany; University of Osnabrueck, GERMANY

## Abstract

Mesaconase catalyzes the hydration of mesaconate (methylfumarate) to (*S*)-citramalate. The enzyme participates in the methylaspartate pathway of glutamate fermentation as well as in the metabolism of various C_5_-dicarboxylic acids such as mesaconate or L-threo-β-methylmalate. We have recently shown that *Burkholderia xenovorans* uses a promiscuous class I fumarase to catalyze this reaction in the course of mesaconate utilization. Here we show that classical *Escherichia coli* class I fumarases A and B (FumA and FumB) are capable of hydrating mesaconate with 4% (FumA) and 19% (FumB) of the catalytic efficiency *k*_*cat*_/*K*_*m*_, compared to the physiological substrate fumarate. Furthermore, the genomes of 14.8% of sequenced *Enterobacteriaceae* (26.5% of *E*. *coli*, 90.6% of *E*. *coli* O157:H7 strains) possess an additional class I fumarase homologue which we designated as fumarase D (FumD). All these organisms are (opportunistic) pathogens. *fumD* is clustered with the key genes for two enzymes of the methylaspartate pathway of glutamate fermentation, glutamate mutase and methylaspartate ammonia lyase, converting glutamate to mesaconate. Heterologously produced FumD was a promiscuous mesaconase/fumarase with a 2- to 3-fold preference for mesaconate over fumarate. Therefore, these bacteria have the genetic potential to convert glutamate to (*S*)-citramalate, but the further fate of citramalate is still unclear. Our bioinformatic analysis identified several other putative mesaconase genes and revealed that mesaconases probably evolved several times from various class I fumarases independently. Most, if not all iron-dependent fumarases, are capable to catalyze mesaconate hydration.

## Introduction

**Fig 1:**
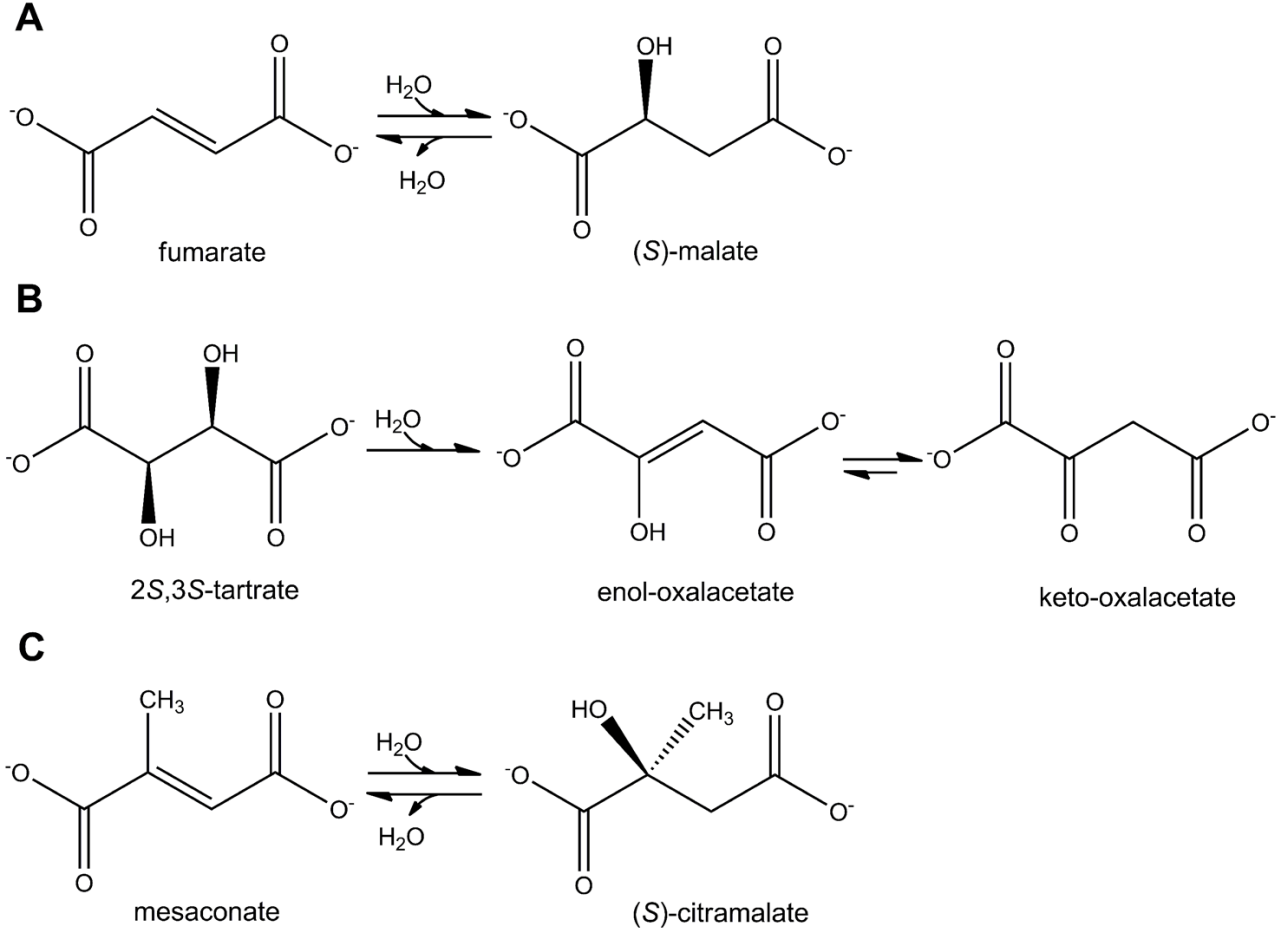
Reactions catalyzed by class I fumarases: (A) fumarase reaction, (B) (2*S*,3*S*)-tartrate dehydratase reaction, and (C) mesaconase reaction.

Fumarase (EC 4.2.1.2) catalyzes the reversible hydration of fumarate to (*S*)-malate (or L-malate in D/L nomenclature) (**Fig 1A**) and participates in the tricarboxylic acid (TCA) cycle and in a number of other metabolic processes. Two phylogenetically unrelated classes of fumarases are known today [1]. The class I enzymes are thermolabile homodimers with a molecular mass of ~120 kDa. They contain an oxygen-sensitive catalytic [4Fe-4S]-cluster acting as a Lewis acid to activate a hydroxyl from the substrate (for elimination) or water (for addition) [2]. The oxidation of the [4Fe-4S]-cluster leads to an inactive [3Fe-4S]-cluster, which can be reactivated upon anaerobic incubation with iron (II) and thiol. Class I fumarases are predominantly found in *Bacteria*, in some *Archaea* [3] and *Eukaryotes*, but not in *Homo sapiens* or *Saccharomyces cerevisiae* [4,5]. *Escherichia coli* possesses two class I enzymes, fumarases A and B (FumA and FumB), sharing a high degree of sequence similarity and having similar catalytic properties [6]. In many *Bacteria* and *Archaea*, fumarase is encoded by two subunits homologous to the N- and C-terminal parts of FumA of *E*. *coli* [7,8]. Class II fumarases like fumarase C (FumC) of *E*. *coli* are thermostable tetramers with four identical 50 kDa subunits that do not require Fe^2+^ for the activity [1]. They are oxygen tolerant enzymes catalyzing (*S*)-malate dehydration through the intermediate formation of an aci-carboxylate and can be found in many pro- and eukaryotic organisms [9].

Class I and class II enzymes have very similar catalytic properties towards fumarate, and it is therefore surprising that many bacteria possess multiple fumarase genes (e.g., three in *E*. *coli* K-12 [1], two in *Burkholderia xenovorans* [10] and *Bacillus stearothermophilus* [11]). The presence of different fumarases should confer selective advantage for the cell, and it was proposed that “each fumarase apparently has a distinct function for which it is particularly well suited” [12]. *E*. *coli* fumarases are controlled in a hierarchical manner depending on the oxidative conditions that the cell encounters: fumarase A is the major fumarase under microoxic conditions, fumarase B under anoxic conditions, and fumarase C under oxic conditions [13]. Furthermore, it was shown that FumB is capable to convert (2*S*,3*S*)-tartrate (D-(-)-tartrate in D/L nomenclature) to oxaloacetate (**Fig 1B**) and is used by *E*. *coli* for (2*S*,3*S*)-tartrate utilization [6,14]. Apart from fumarate and (2*S*,3*S*)-tartrate, *E*. *coli* FumA is capable to hydrate acetylene dicarboxylate and fluorofumarate [12], although these reactions are probably not of physiological importance.

**Fig 2:**
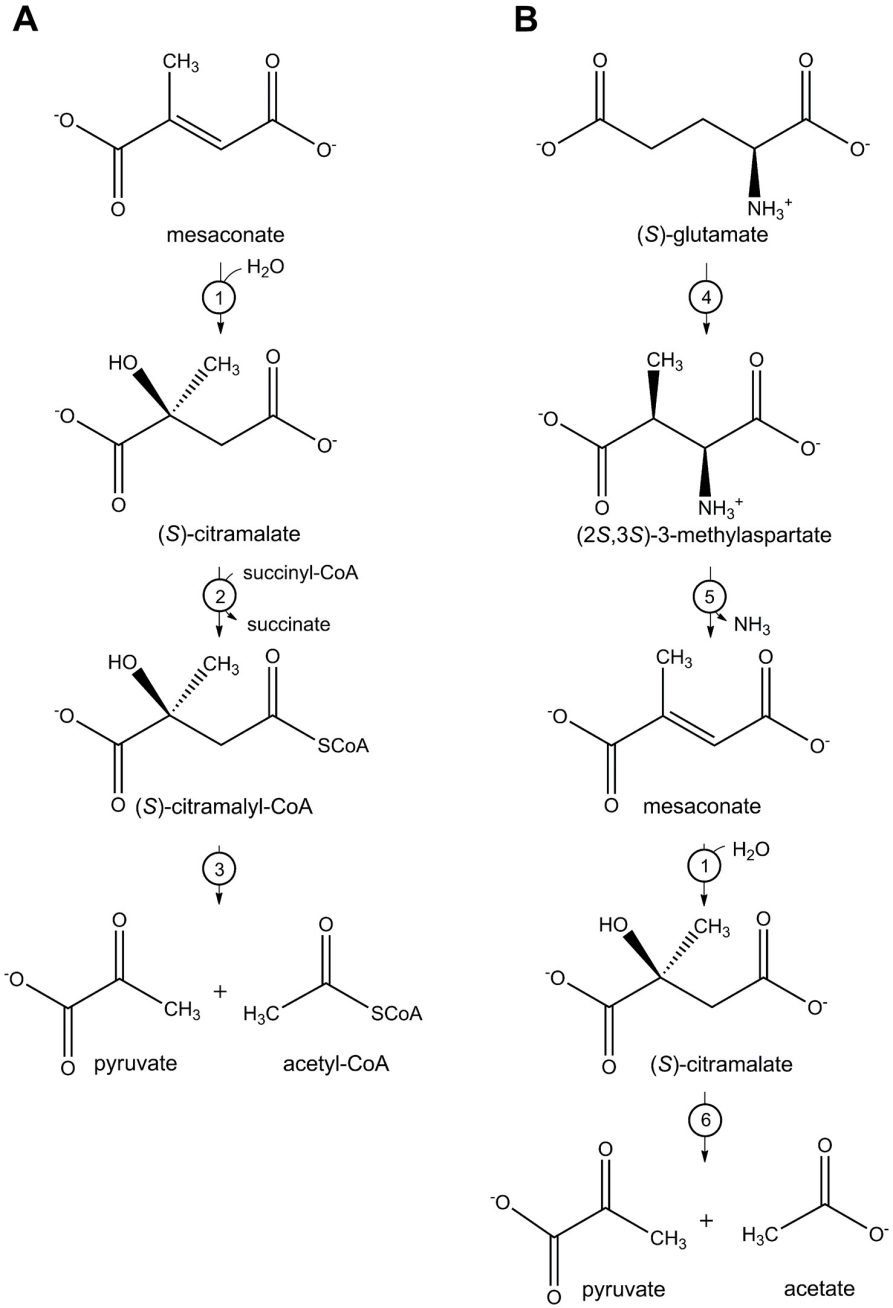
Metabolic pathways involving mesaconase reaction: (A) mesaconate utilization, (B) methylaspartate pathway of glutamate fermentation. Enzymes: 1, mesaconase; 2, succinyl-CoA:(*S*)-citramalate CoA-transferase; 3, (*S*)-citramalyl-CoA lyase; 4, glutamate mutase; 5, methylaspartate ammonia-lyase; 6, citramalate lyase.

We have recently shown that *B*. *xenovorans* class I fumarase is equally efficient in the hydration of fumarate to (*S*)-malate and mesaconate to (*S*)-citramalate (L-citramalate in D/L nomenclature) (**Fig 1C**) and participates in mesaconate utilization in this bacterium (**Fig 2**) [10]. Despite the high similarity of fumarate and mesaconate (methylfumarate), the finding was unexpected, as homologous class I fumarases of *E*. *coli* and *Syntrophobacter fumaroxidans* were already tested for mesaconase activity, and the obtained results were negative [1,12,15,16].

Mesaconase was first identified in the methylaspartate pathway of glutamate fermentation in *Clostridia*. In this pathway, glutamate is converted in the B_12_-dependent glutamate mutase reaction to methylaspartate, which is deaminated to mesaconate by methylaspartate ammonia-lyase. Mesaconase further converts mesaconate to (*S*)-citramalate, which is cleaved by citramalate lyase to pyruvate and acetate. Mesaconase from *Clostridium tetanomorphum* was purified and characterized in the 1960^th^ and was shown to be an oxygen-sensitive and Fe-dependent heterodimer possessing also fumarase activity [17,18,19]. Furthermore, the participation of mesaconase in acetate assimilation in *Rhodospirillum rubrum* was proposed [20,21], although the corresponding pathway still needs to be proven [22]. Mesaconase from a soil *Bacillus* sp. participates in L-threo-β-methylmalate and mesaconate metabolism [23,24] and has properties similar to those of class I fumarase from *Burkholderia* [10]. These data suggest that the presence of mesaconase activity may be a general feature of class I fumarases, being in line with the notion that enzymes, transporters and sensors interacting with different C_4_-dicarboxylates have often a broad specificity over their substrate (e.g., [25])

In this work, we have cloned and expressed the genes of *E*. *coli* fumarases A and B and characterized the corresponding proteins. We show that these enzymes are promiscuous catalyzing also mesaconate hydration with 10% of catalytic efficiency, compared to fumarate. Furthermore, we have found that most of enterohemorrhagic *E*. *coli* (EHEC) strains possess an additional gene for class I fumarase. It is clustered with genes for the key enzymes of the methylaspartate pathway of glutamate fermentation and encodes a hydratase highly specific for mesaconase as a substrate. Our bioinformatic analysis suggests that mesaconases in other species also belong to class I fumarases or are phylogenetically related to them.

## Materials and Methods

### Materials

Chemicals were obtained from Fluka (Neu-Ulm, Germany), Sigma-Aldrich (Deisenhofen, Germany), Merck (Darmstadt, Germany), Serva (Heidelberg, Germany), or Roth (Karlsruhe, Germany). Biochemicals were from Roche Diagnostics (Mannheim, Germany), AppliChem (Darmstadt, Germany), or Gerbu (Craiberg, Germany). Materials for cloning and expression were purchased from MBI Fermentas (St. Leon-Rot, Germany), New England BioLabs (Frankfurt, Germany), Novagen (Schwalbach, Germany), Genaxxon Bioscience GmbH (Biberach, Germany), Biomers (Ulm, Germany), or Qiagen (Hilden, Germany). Materials and equipment for protein purification were obtained from GE Healthcare (Freiburg, Germany), Macherey-Nagel (Düren, Germany), Pall Corporation (Dreieich, Germany) or Millipore (Eschborn, Germany).

### Microbial strains and growth conditions

*Escherichia coli* strains K12 W3110 (DSMZ 5911) and ATCC 700728 (DSMZ 17076) were obtained from Deutsche Sammlung von Mikroorganismen und Zellkulturen (DSMZ). The *E*. *coli* K-12 AG1 strains containing the respective plasmid-encoded fumarase genes *fumA* (strain JW1604), *fumB* (strain JW4083) and *fumC* (strain JW1603) from *E*. *coli* K-12 W3110 were obtained from the *E*. *coli* ASKA library [26]. The growth of the K-12 strain was tested in the presence of mesaconate (20 mM) under both oxic and anoxic conditions in the minimal medium described Kronen et al. [10]. For some experiments, the cells of the ATCC 700728 strain were grown under oxic conditions at 37°C in the same minimal medium with acetate (50 mM) or propionate (50 mM) and citrate (2 mM). Furthermore, we attempted to grow the ATCC 700728 strain in this minimal medium under anaerobic conditions in the presence of glutamate (80 mM) or yeast extract (0.5%) plus glutamate (80 mM) as well as in the glutamate/yeast extract medium described by Kato and Asano [27]. In addition, we grew these cells in the lysogeny broth (LB) medium (bactotryptone, 10 g l^-1^, yeast extract, 5 g l^-1^, NaCl, 10 g l^-1^) and in the LB-glutamate (80 mM) medium. All anaerobic growth tests were done in the presence and in the absence of cobalamins. Three different forms of cobalamin were tested: hydroxocobalamin (0.2 and 2 μM), cyanocobalamin (0.2 and 2 μM) and adenosylcobalamin (0.2 and 2 μM).

### Preparation of cell extracts

For the cell extract enzyme assays, cell extracts were prepared under oxic conditions using a mixer-mill (MM200, Retsch, Haare, Germany). Cells (100–150 mg) were suspended in 0.5 ml of 20 mM 3-(N-morpholino)propanesulfonic acid (Mops)/KOH, pH 6.9, 0.1 mg ml^-1^ DNase I and 0.5 mM dithiothreitol (DTT) in 2.2 ml Eppendorf vials. Then, 1 g of glass beads (diameter 0.1–0.25 mm) was added to the suspension, and the cells were treated in the mixer-mill for 10 min at 30 Hz. This was followed by a centrifugation step (14,000 g, 4°C, 10 min), and the supernatant (cell extract) was used for enzyme assays. The protein content of the cell extract was 6 to 20 mg ml^-1^.

### Enzyme assays

Spectrophotometric enzyme assays were performed in 0.5 ml cuvettes at 37°C, unless otherwise indicated. Anaerobic assays were done in an anaerobic chamber. One unit of enzyme activity (U) corresponds to 1 μmol substrate converted per minute.

*Fumarase* and *mesaconase* activities were measured under anoxic (class I enzymes) or oxic (fumarase C) conditions in quartz cuvettes spectrophotometrically at 240 nm (fumarase, ε_fumarate_ = 2.4 mM^-1^ cm^-1^ [12]) or 250 nm (mesaconase; ε_mesaconate_ = 2.26 mM^-1^ cm^-1^ [17]), as described previously [10]. The typical assay mixture contained 100 mM MOPS/KOH (pH 6.9), 5 mM MgCl_2_, 5 mM DTT, 0.4 mM fumarate/mesaconate (for forward reaction) or 5 mM (*S*)-malate/(*S*)-citramalate (for reverse reaction), and 0.02–1 μg reactivated fumarases. In addition, (*R*)-malate (20 mM) and (*R*)-citramalate (20 mM) (D-malate and D-citramalate in D/L nomenclature) were tested as the substrates of fumarases. For these assays, 10–20 μg reactivated fumarases was used.

*Tartrate dehydratase* activity of fumarases was determined under anoxic (class I enzymes) or oxic (fumarase C) conditions as tartrate-dependent oxaloacetate formation by coupling the reaction to malate dehydrogenase and following NADH oxidation at 365 nm (ε_NADH_ = 3.4 mM^-1^ cm^-1^ [28]). The assay mixture contained 100 mM MOPS/KOH (pH 6.9), 5 mM DTT, 0.25 mM NADH, 6 U malate dehydrogenase (Sigma), 8–50 μg reactivated fumarases, and varying concentrations of (2*S*,3*S*)-tartrate from 0.5–25 mM. Furthermore, (2*R*,3*R*)-tartrate (L-(+)-tartrate in D/L nomenclature) (7 mM) and *meso*-tartrate (7 mM) were tested as the substrates of fumarases.

*Citrate lyase* was measured under oxic conditions spectrophotometrically in a malate dehydrogenase coupled assay. The assay mixture contained 100 mM Tris/HCl (pH 7.8), 5 mM DTT, 0.5 mM NADH, 6 U malate dehydrogenase (Sigma), cell extract (40–100 μg protein) and 20 mM citrate.

*Citramalate lyase* was measured as described for citrate lyase, but with (*S*)-citramalate and lactate dehydrogenase (Sigma) instead of citrate and malate dehydrogenase.

*Isocitrate lyase* was assayed under oxic conditions spectrophotometrically as isocitrate-dependent glyoxylate formation in the presence of phenylhydrazine at 324 nm (ε_glyoxylate phenylhydrazone_ = 17 mM^-1^ cm^-1^ [29]). The assay mixture contained 100 mM MOPS/KOH (pH 6.9), 5 mM MgCl_2,_ 5 mM DTT, 3.5 mM phenylhydrazine, 4 mM isocitrate, and cell extract (15–30 μg protein).

*Methylcitrate synthase* activity was measured under oxic conditions spectrophotometrically at 412 nm following oxaloacetate-dependent formation of CoA from propionyl-CoA in the presence of the 5,5'-dithiobis-(2-nitrobenzoic acid) (DTNB; ε_DTNB-CoA_ = 14.2 mM^-1^ cm^-1^ [30]). The assay mixture contained 100 mM MOPS/KOH (pH 6.9), 5 mM MgCl_2,_ 1 mM DTNB, 0.5 mM propionyl-CoA, 5 mM oxaloacetate, and cell extract (20–100 μg protein).

*Methylaspartate ammonia-lyase* activity was measured under aerobic conditions either spectrophotometrically by measuring the methylaspartate-dependent formation of mesaconate at 250 nm [31], or in a high-performance liquid chromatography (HPLC)-based assay [32]. The reaction mixture for the spectrophotometric enzyme assay consisted of 50 mM ethanolamine/HCl buffer (pH 9.7), 10 mM KCl, 1 mM MgCl_2,_ 5 mM methylaspartate, and cell extract. For the measurement in the HPLC-based assay, the reaction mixture (0.5 ml) contained 100 mM MOPS buffer (pH 6.9) 50 mM DTT, 5 mM MgCl_2_, and 0.05 mM methylaspartate. The reaction was started with cell extract (140 μg protein). Samples (100 μl) were withdrawn at different time points (0, 10, 60 min) and mixed with 50 μl 1 M HCl to stop the reaction. After centrifugation (20 min, 4°C, 20,000 g), the samples were diluted (1:2) with H_2_O and analyzed by HPLC, as described previously [32].

### Reactivation of fumarases

As exposure to oxygen (partly) inactivated the FeS clusters of class I fumarases, the enzymes had to be reactivated by activation with DTT and Fe^2+^. The reactivation was performed, as described previously [10].

### Cloning of fumarase D genes from *E*.*coli* ATCC 700728

The *fumD* gene (*ecatcc700728_0849*) was amplified by PCR from *E*.*coli* O157:H7 ATCC 700728 chromosomal DNA with the Q5 High-Fidelity DNA polymerase (New England Biolabs) according to manufacturer’s instructions with the following primers: FumD_fwd (CATATCGAAGGTCGTCATATGTCCAAACCATTTATCTGGC), and FumD_rev (GACAGCTTATCATCGATATTAATGTCCCGCAGGGCA). Primers were constructed with NEB Builder (http://nebuilder.neb.com/) and contained adapter sequences for the cloning vector pET16b (underlined) and NdeI restriction site (in bold). Thirty-four cycles were conducted in an automated thermocycler under the following conditions: a 20 s (3 min in the first cycle) denaturation at 98°C, annealing for 30 s at 63.8°C, and primer extension for 1.5 min (5 min in the final cycle) at 72°C. The PCR products were purified and ligated into the pET16b vector (restricted with HindIII and NdeI), using the Gibson Assembly Cloning Kit (New England Biolabs, Frankfurt) according to the manufacturer’s protocol.

### Homologous expression of fumarases in *E*. *coli*

The *E*. *coli* K-12 AG1 strains containing the overexpressed *fumA*, *fumB* and *fumC* were grown aerobically at 37°C in LB medium supplemented with chloramphenicol (34 μg ml^-1^), 100 μM FeSO_4_ and 100 μM Fe(II)citrate. Expression was induced with 0.15 mM isopropyl-thio-β-D-galactoside (IPTG) as the cells reached an optical density at 578 nm (OD_578_) of 0.4–0.5. After additional growth for 3 h at 37°C, cells were harvested by centrifugation (4,500 × g, 10 min, 4°C) and stored at –20°C until use.

Fumarase D was produced in *E*. *coli lysY* cells (New England Biolabs, Frankfurt) that had been transformed with the corresponding plasmid. The cells were grown aerobically at 37°C in LB medium supplemented with 100 μg ml^-1^ ampicillin, 100 μM FeSO_4_ and 100 μM Fe(II)citrate. Expression was induced at OD_578_ of 0.4–0.5 with 0.8 mM IPTG. After the induction, the culture was transferred to a sterile Schott bottle that was shut tightly for anaerobic conditions and slightly shaken at 20°C. After additional growth for 12–15 h, the cells were harvested under anaerobic conditions and stored in an anaerobic Schott bottle at -20°C until use.

### Purification of recombinant enzymes

Frozen *E*. *coli* cells from the respective homologously expressed fumarases were suspended in a buffer containing 40 mM Tris/HCl (pH 7.8), 0.1 mg ml^-1^ DNase I and 0.5 mM DTT (1.5 ml resuspension buffer per 1 g cell mass). The suspensions were passed twice trough a chilled French pressure cell at 137 MPa, and the cell lysate was centrifuged for 1 h at 100,000 × g and 4°C. The supernatant was loaded onto a 1 ml Protino Ni-NTA column (Macherey-Nagel) previously equilibrated with buffer A (50 mM Tris/HCl, pH 7.8, 300 mM NaCl, 5 mM MgCl_2_). Purification was carried at a flow rate of 1 ml min^-1^. The His-tagged proteins were eluted at different concentrations of imidazole (25, 50, 300, 500 mM) obtained by mixing buffer A with buffer B (buffer A with 500 mM imidazole). The enzymes were concentrated by ultrafiltration (Amicon YM 10 membrane, Millipore) and stored at -20°C with 50% (v/v) glycerol. For fumarase D, all purifications steps had to be performed under anaerobic conditions.

### Molecular biological techniques

The *in silico* cloning steps were performed with the program Clone Manager 7.11 (Scientific & Educational Software). Standard protocols were used for purification, preparation, cloning, transformation and amplification of DNA [33,34,35]. Plasmid DNA isolation, purification of PCR products and plasmids were performed using Qiagen kits according to the manufacturer’s specifications.

### Database search and phylogenetic analysis

Query sequences were obtained from the National Center for Biotechnology Information (NCBI) database. The BLAST searches were performed via NCBI BLAST server (http://www.ncbi.nlm.nih.gov/BLAST/) [36]. The amino acid sequences were aligned with sequences from GenBank using CLUSTALW [37] implemented within BioEdit software (http://www.mbio.ncsu.edu/BioEdit/bioedit.html). The phylogenetic tree was reconstructed using a neighbor-joining algorithm [38] in the TREECONW program package [39]. The positions with gaps were not taken into account in the phylogenetic reconstructions.

### Other methods

DNA sequence determination of purified plasmids was performed by GATC Biotech (Konstanz, Germany). SDS-polyacrylamide gel electrophoresis (SDS/PAGE; 12.5%) was performed using the Laemmli method [40]. Proteins were visualized by Coomassie blue staining [41]. Protein concentration was measured according to the Bradford method [42], using BSA as standard.

## Results

### Characterization of fumarases A, B and C from *E*. *coli*

**Fig 3:**
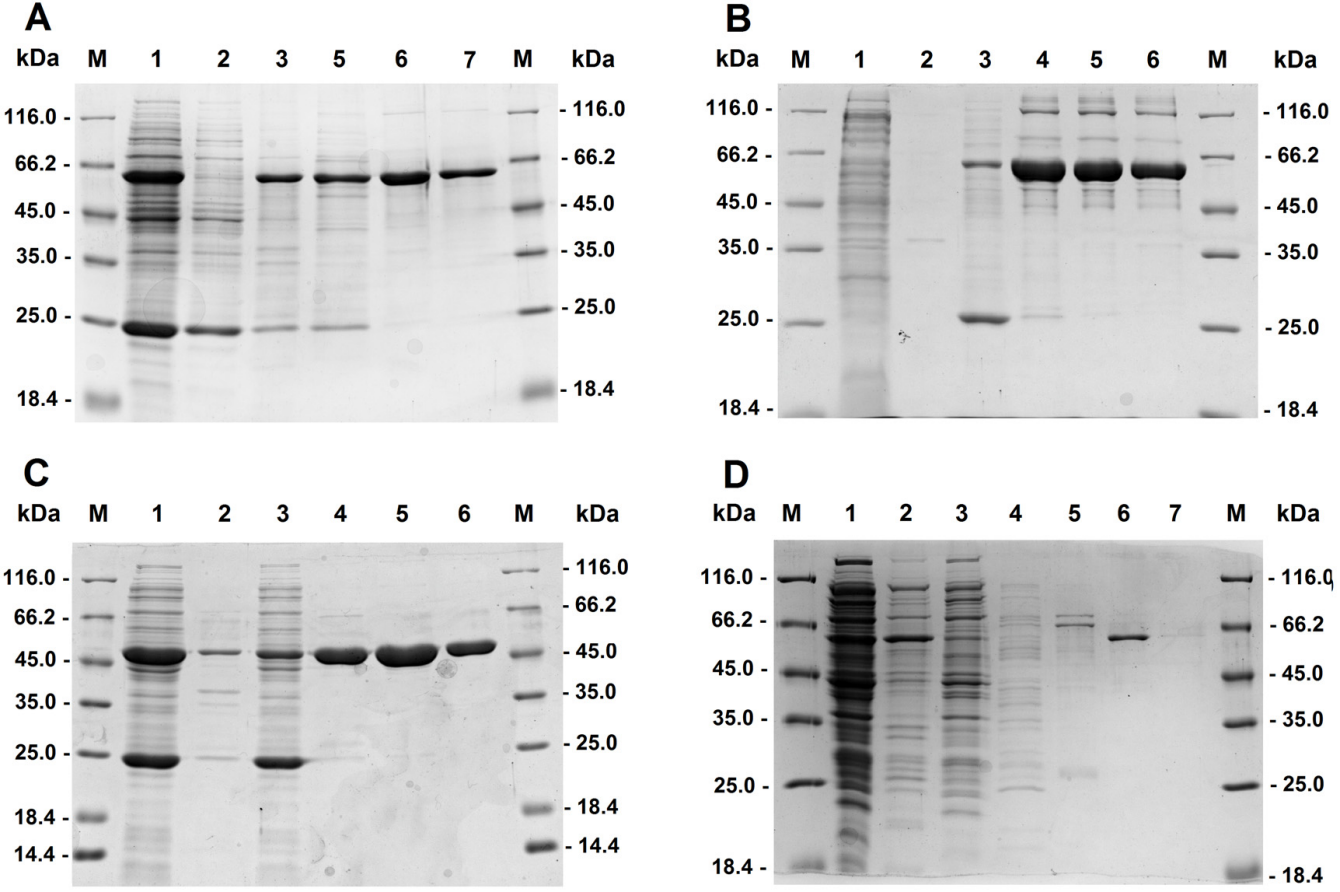
SDS-PAGE (12.5%) of fractions obtained during purification of recombinant fumarases FumA (A), FumB (B) and FumC (C) from *E*. *coli* K-12 as well as of a recombinant mesaconase/fumarase FumD from *E*. *coli* O157:H7 (ATCC 700728) (D). M, molecular mass standard proteins; lane 1, cell extract of *E*. *coli* producing the corresponding proteins; lane 2, membrane fraction; lane 3, flow through after Ni-NTA column; lane 4, elution with 25 mM imidazole; lane 5, elution with 50 mM imidazole; lane 6, elution with 300 mM imidazole; lane 7 with 500 mM imidazole. The fractions eluted with 300 mM imidazole were used for the following enzyme characterization. Proteins were stained with Coomassie blue. The predicted molecular mass of native FumA is 60.3 kDa, that of FumB 60.1 kDa, that of FumC 50.5 kDa, and that of FumD 60.1 kDa.

To test the activities of fumarases of a classical *E*. *coli* K-12 strain (W3110) with mesaconate, we homologously produced class I fumarases FumA and FumB as well as a class II fumarase FumC. The corresponding plasmids were obtained from the ASKA library [26]. The proteins containing N-terminal His-tags were aerobically purified using a Ni-NTA column. SDS-PAGE analysis revealed the presence of a major band with an apparent molecular mass of 60 kDa (for FumA and FumB) and 50 kDa (for FumC) in the 50 mM (for FumA) or 300 mM imidazole fractions (for FumB and FumC) (**Fig 3**), which were used to study the catalytic properties of the enzymes.

All three enzymes catalyzed the reversible conversion of fumarate into (*S*)-malate with very similar catalytic efficiencies *k*_*cat*_/*K*_*m*_ (**Table 1**). Class I enzymes were oxygen-sensitive and required anoxic conditions for maximal activity; incubation of the enzyme with Fe^2+^ and thiol restored full enzymatic activity. On the opposite, class II fumarase FumC remained stable under oxic conditions, and its activity did not increase upon incubation with Fe^2+^ and DTT. All three enzymes were active with (2*S*,3*S*)-tartrate, but much less than with (*S*)-malate (**Table 1**). FumB had the highest catalytic efficiency with this substrate, corroborating the published results [6] and the requirement of FumB for (2*S*,3*S*)-tartrate metabolism [14]. The enzymes were not able to convert (*R*)-malate, (2*R*,3*R*)-tartrate, *meso*-tartrate or (*R*)-citramalate. Unlike FumC, both class I fumarases catalyzed the reversible conversion of mesaconate into (*S*)-citramalate, with FumB being a better catalyst for mesaconase reaction (10/19% of catalytic efficiency with (*S*)-citramalate/mesaconate, compared to (*S*)-malate/fumarate) (**Table 1**). The catalytic efficiency of FumA with (*S*)-citramalate/mesaconate was ~4% of that with fumarate or (*S*)-malate.

**Table 1:** Catalytic properties of recombinant fumarases from *E*. *coli* K-12 W3110.

Enzyme	Substrate	*V*_*max*_ (U mg^-1^ protein)	*K*_*m*_ (mM)	*k*_*cat*_/*K*_*m*_ (M^-1^ s^-1^)
Class I fumarase A (BAA15360)	Fumarate	614 ± 29	0.094 ± 0.015	6.6 × 10^6^
	(*S*)-Malate	350 ± 10	0.4 ± 0.05	8.7 × 10^5^
	Mesaconate	55.6 ± 1.7	0.22 ± 0.02	2.5 × 10^5^
	(*S*)-Citramalate	37.4 ± 1.4	1.08 ± 0.13	3.5 × 10^4^
	(*S*,*S*)-(-)-Tartrate	1.4 ± 0.04	2 ± 0.2	635
Class I fumarase B (BAE78124)	Fumarate	654 ± 38	0.21 ± 0.03	3.12 × 10^6^
	(*S*)-Malate	289 ± 16	0.78 ± 0.13	3.7 × 10^5^
	Mesaconate	57.8 ± 1.4	0.1 ± 0.007	5.8 × 10^5^
	(*S*)-Citramalate	39.4 ± 1.3	1.04 ± 0.11	3.8 × 10^4^
	(*S*,*S*)-(-)-Tartrate	1.7 ± 0.04	0.80 ± 0.05	2130
Class II fumarase C (BAA15349)	Fumarate	1164 ± 55	0.28 ± 0.03	3.5 × 10^6^
	(*S*)-Malate	431 ± 11	0.93 ± 0.12	3.9 × 10^5^
	Mesaconate	< 0.1	-	-
	(*S*)-Citramalate	< 0.1	-	-
	(*S*,*S*)-(-)-Tartrate	0.48 ± 0.06	6.2 ± 2.1	65

Values are means ± standard deviations of results from at least three measurements.

### Identification of putative mesaconase genes in bacteria possessing the methylaspartate pathway for glutamate fermentation

As the methylaspartate pathway of glutamate fermentation involves mesaconase reaction (**Fig 2**), we decided to check the presence of class I fumarase homologues in the vicinity of the genes for glutamate mutase and methylaspartate ammonia-lyase in the genomes of bacteria possessing both of these enzymes (as described in [32]).

**Fig 4:**
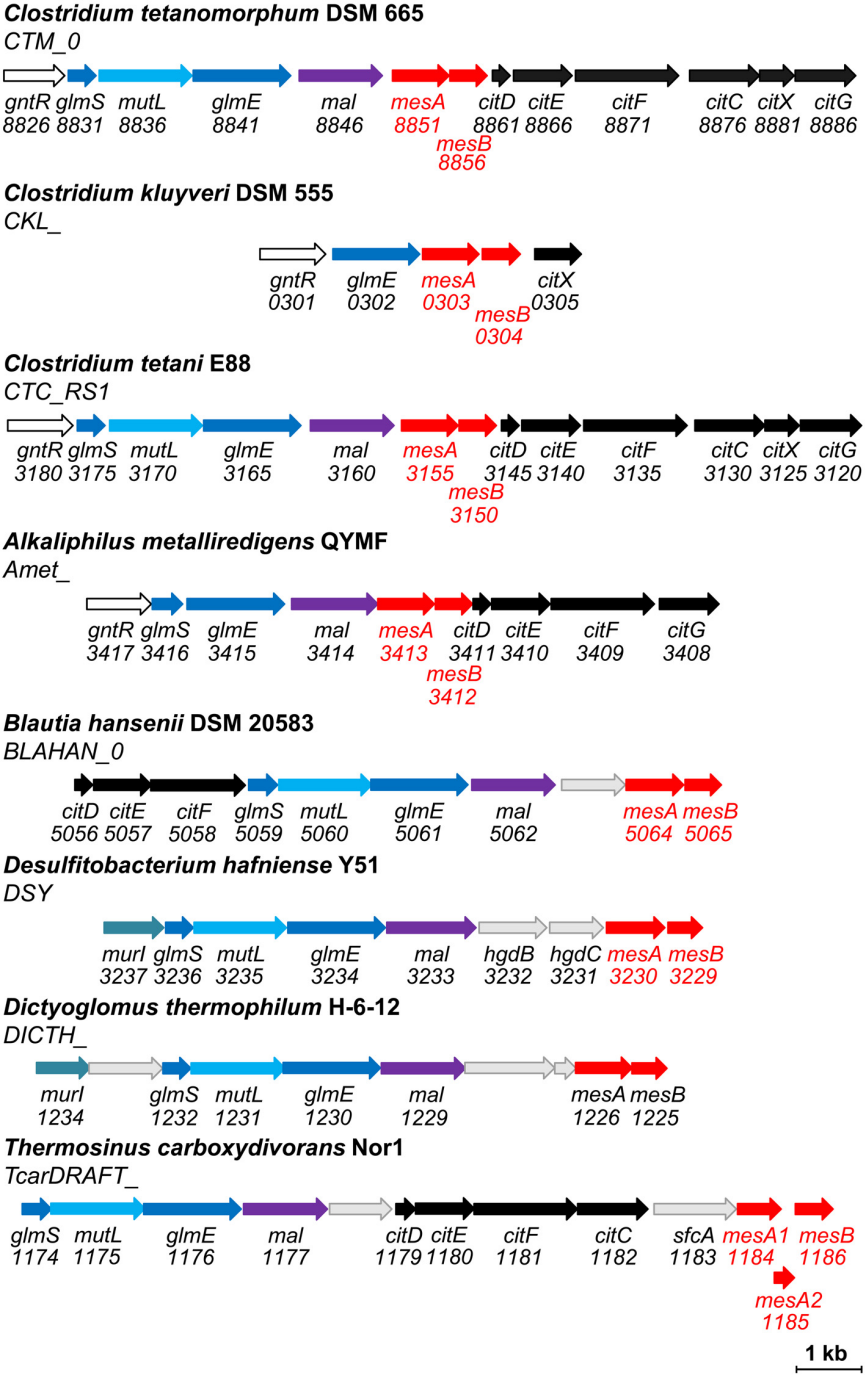
Co-localization of the genes for methylaspartate ammonia-lyase and glutamate mutase with the gene(s) for class I fumarase (mesaconase). GntR, a putative transcriptional regulator for glutamate degradation; GlmS and GlmE, subunits of glutamate mutase; MutL (sometimes designated also as GlmL), a chaperone involved in reactivation of glutamate mutase [46]; Mal, methylaspartate ammonia-lyase; MesAB, α- and β-subunits of a putative mesaconase; CitDEF and CitCXG, subunits of citramalate lyase and enzymes involved in its biosynthesis/activation (named after the subunits of homologous citrate lyase [47,48]); MurI, glutamate racemase; HgdBC, subunits of 2-hydroxyglutaryl-CoA dehydratase; SfcA, malate dehydrogenase, decarboxylating.

*Clostridium tetanomorphum* as well as many other *Clostridia* possess the gene cluster containing genes for glutamate fermentation (e.g., *C*. *tetani*, *C*. *carboxidivorans*, *C*. *ljungdahlii*, *Thermobrachium celere*). Besides glutamate mutase and methylaspartate ammonia-lyase, this cluster encodes genes for the putative citramalate lyase (annotated as citrate lyase genes; note that the citramalate and citrate lyase reactions are very similar and most probably are catalyzed by homologous enzymes [43]). Furthermore, there are also two genes encoding homologues of N- and C-terminal domains of class I fumarase in this genes cluster (**Fig 4**), which were putatively designated as mesaconase genes for *C*. *tetani* [44]. Similar gene clusters can be found in the genomes of *Blautia hansenii* (*Firmicutes*, *Clostridia*), *Alkaliphilus metalliredigens* (*Firmicutes*, *Clostridia*), *Thermosinus carboxydivorans* (*Firmicutes*, *Negativicutes*), *Dictyoglomus thermophilum* (*Dictyoglomi*), *Desulfitobacterium hafniense* (*Firmicutes*, *Clostridia*) (**Fig 4**). In *Treponema denticola* (*Spirochaetes*), class I fumarase genes are located together with citrate lyase genes, whereas methylaspartate ammonia-lyase and glutamate mutase genes are located elsewhere in the genome. In *Clostridium kluyveri*, only a rudimentary glutamate fermentation gene cluster is present (**Fig 4**), which contains, apart from a putative mesaconase gene, only genes encoding one of the subunits of glutamate mutase and an enzyme involved in citrate (citramalate) lyase biogenesis. Some *Archaea* (haloarchaea) possess genes for glutamate mutase and methylaspartate ammonia-lyase as well. However, these enzymes participate in haloarchaea in the methylaspartate cycle of acetate assimilation rather than in glutamate fermentation. In this cycle, mesaconate formed in the methylaspartate ammonia-lyase reaction is metabolized through the activation to mesaconyl-CoA rather than via the mesaconase reaction [32,45].

**Fig 5:**
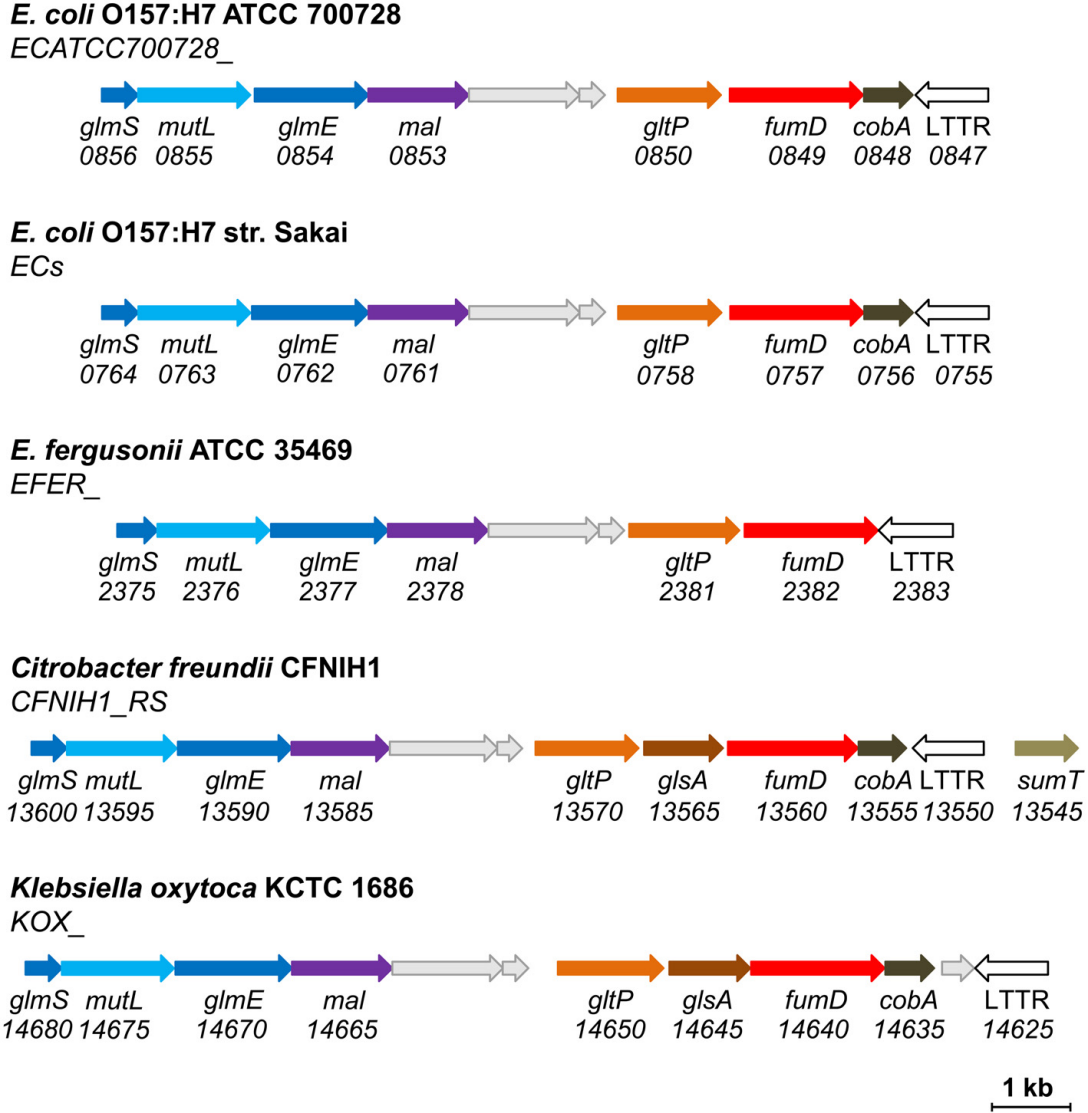
Co-localization of the genes for methylaspartate ammonia-lyase and glutamate mutase with mesaconase gene in *Enterobacteriaceae*. GltP, glutamate transporter; FumD, promiscuous mesaconase/fumarase; CobA, cob(I)alamin adenosyltransferase; LTTR, a putative LysR family transcriptional regulator; GlsA, glutaminase; SumT, uroporphyrinogen-III C-methyltransferase; for the other abbreviations, see **Fig 4**. Note that the gene encoding a protein of unknown function (DUF1446) downstream of *mal* is often annotated as methylaspartate ammonia-lyase, for unknown reason. A homologous gene is present downstream of *mal* in *Dictyoglomus thermophilum* (**Fig 4**). Note also that glutaminase is known to contribute to the acid resistance that is an important feature for enterobacterial virulence [49,50].

Interestingly, 14.8% of all sequenced *Enterobacteriaceae* strains (26.5% of all *E*. *coli* strains, 90.6% of *E*. *coli* O157:H7) possess the genes for methylaspartate ammonia-lyase and glutamate mutase. In all these cases, these genes are co-localized with an additional copy of class I fumarase (fourth fumarase gene in *E*. *coli* O157:H7) (**Fig 5**). We designated the corresponding protein as fumarase D (FumD) and decided to characterize it biochemically.

### Characterization of fumarase D from *E*. *coli* O157:H7

The *fumD* gene from *E*. *coli* O157:H7 (ATCC 700728) was cloned and expressed in *E*. *coli lysY*. The resulting protein was purified using a Ni-NTA column and analyzed using SDS-PAGE. A major band with an apparent molecular mass of ~60 kDa was revealed in the 300 mM imidazole fraction (**Fig 3**). Aerobic purification resulted in inactive protein, which could only partly be reactivated with Fe^2+^ and thiol (38 U mg^-1^ protein, measured as mesaconate hydration). However, if all operations (e.g., protein expression, purification, reactivation and enzyme assays) were performed under anoxic conditions, significantly higher enzymatic activity could be measured (**Table 2**). In fact, the enzyme has higher catalytic efficiency *k*_*cat*_/*K*_*m*_ for mesaconate than for fumarate and therefore appears to be a true mesaconase rather than fumarase. The clustering of *fumD* with methylaspartate ammonia-lyase and glutamate mutase genes (**Fig 5**) implies the involvement of FumD in glutamate conversion to (*S*)-citramalate.

**Table 2:** Catalytic properties of recombinant fumarase D (ECs0757; ECATCC700728_0849) from *E*. *coli* O157:H7 (ATCC 700728).

Substrate	*V*_*max*_ (U mg^-1^ protein)	*K*_*m*_ (mM)	*k*_*cat*_/*K*_*m*_ (M^-1^ s^-1^)
Fumarate	671 ± 28	0.9 ± 0.1	7.5 × 10^5^
(*S*)-Malate	52 ± 2	0.2 ± 0.025	2.6 × 10^5^
Mesaconate	292 ± 13	0.15 ± 0.02	2 × 10^6^
(*S*)-Citramalate	104 ± 4	0.92 ± 0.10	1.1 × 10^5^
(*S*,*S*)-(-)-Tartrate	0.13 ± 0.01	2.6 ± 0.6	50

Values are means ± standard deviations of results from at least three measurements.

### Presence of glutamate fermentation gene cluster in pathogenic enterobacteria

All enterobacteria carrying this cluster appear to be (opportunistic) pathogens. Indeed, homologs of *E*. *coli* O157:H7 glutamate fermentation gene cluster can be found in e.g. enteropathogenic *E*. *coli* O55:H7 strains, shigatoxigenic *E*. *coli* O145:H28 strains, *Klebsiella oxytoca*, *Citrobacter freundii*, *Enterobacter cloacae*, *Shigella flexneri*, *Salmonella enterica*, *E*. *fergusonii*, *Yersinia enterocolitica*, *Morganella morganii*, among others. The presence of the glutamate fermentation cluster in these pathogenic species suggests a possible role of glutamate fermentation in pathogenicity. Glutamate is known to be present in the intestinal tract in considerable amounts (of millimolar range [51]) and could therefore serve as an important carbon and energy source for pathogens. For that, (*S*)-citramalate should be further converted to pyruvate and acetate/acetyl-CoA via citramalate or citramalyl-CoA lyase reactions. As most enterobacteria do not possess citramalyl-CoA lyase genes [52], we first suggested that the citramalate cleavage proceeds through the activity of citrate lyase which is present in many *Enterobacteriaceae* [53].

### Fate of (*S*)-citramalate produced from glutamate

*E*. *coli* ATCC 700728 possesses a set of genes encoding citrate lyase which, taking into account the similarity of citrate and citramalate lyases, may catalyze both reactions. In order to test this hypothesis, we grew *E*. *coli* ATCC 700728 in the presence of citrate and tested it for citrate and citramalate lyase activities. Although we measured high activity of citrate lyase under these conditions (0.48 ± 0.05 U mg^-1^ protein), no activity of citramalate lyase could be detected. Further possible candidates for catalyzing the citramalate cleavage were isocitrate lyase (0.79 ± 0.04 U mg^-1^ protein in acetate-grown cells) and methylisocitrate lyase (involved in the methylcitrate pathway of propionate assimilation in *E*. *coli* [54,55]; the activity of the key enzyme of the pathway, methylcitrate synthase, was 0.62 ± 0.10 U mg^-1^ protein in propionate-grown cells). However, no citramalate lyase activity could be detected in both acetate- and propionate-grown cells (measurement limit: 1 mU mg^-1^ protein). Therefore, the fate of (*S*)-citramalate produced from glutamate is still unclear.

All our attempts to find conditions under which *E*. *coli* ATCC 700728 expresses the glutamate fermentation genes failed. No growth was detected with glutamate as a sole carbon source under anoxic conditions, and glutamate did not stimulate anaerobic growth of *E*. *coli* ATCC 700728 on the LB medium. Furthermore, we could not detect any activity of methylaspartate ammonia-lyase in these cells (measurement limit: 0.1 mU mg^-1^ protein), suggesting that the enzyme is not produced under these conditions. Previously, the activity of methylaspartate ammonia-lyase was shown to be widespread among enterobacteria, although none of the strains was capable to grow on glutamate or methylaspartate alone [27,56]. However, *E*. *coli* ATCC 700728 strain cultivated under the same conditions as by Kato and Asano [27] did not possess any detectable activity of this enzyme either (data not shown).

### Phylogenetic analysis of class I fumarases

**Fig 6:**
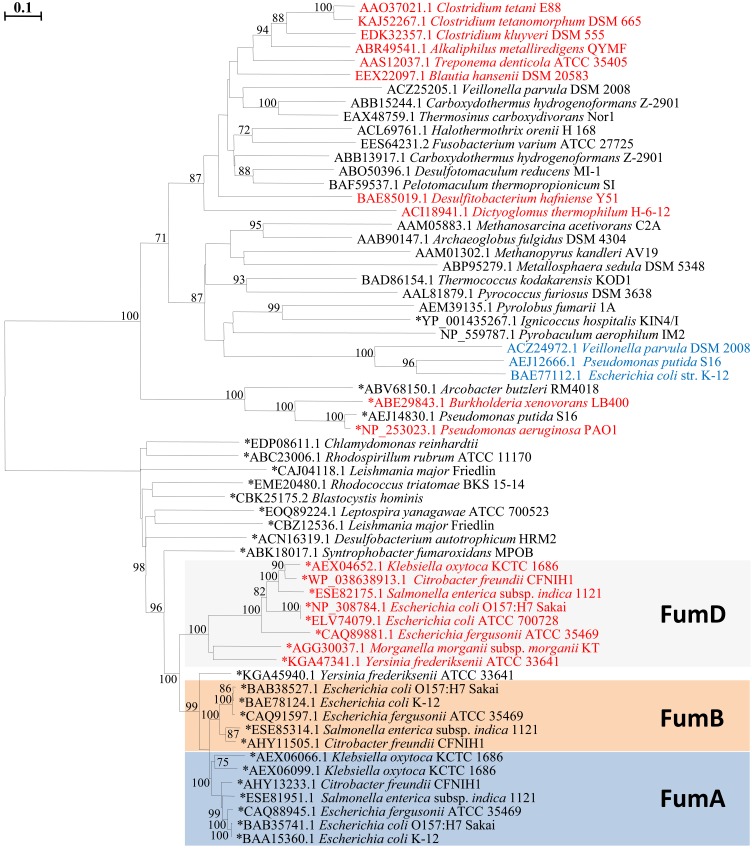
Phylogenetic tree of class I fumarases and related proteins. The tree is based on amino acid sequence analysis of N-terminal part of class I fumarases consisting of one subunit (marked with”*” on the tree) and α-subunits of heterodimeric fumarases/ mesaconases/ (2*R*,3*R*)-tartrate dehydratases. (2*R*,3*R*)-Tartrate dehydratases are shown in blue, enzymes functioning *in vivo* as mesaconases in red (functions confirmed experimentally or based on the co-localization with glutamate fermentation genes). Tree topography and evolutionary distances are given by the neighbor-joining method with Poisson correction. The scale bar represents a difference of 0.1 substitutions per site. Numbers at nodes indicate the percentage bootstrap values for the clade of this group in 1000 replications. Only values above 70% were considered.

Data base search showed that the sequences identified as (potential) mesaconases are distributed along different phylogenetic clusters in the tree (**Fig 6**). Heterodimeric class I fumarases and mesaconases are located in one cluster with the genes encoding (2*R*,3*R*)-tartrate dehydratases, with the latter being most closely related to the archaeal proteins. These data imply that mesaconase evolved from various class I fumarases several times during the evolution. Together with intrinsic mesaconase activity of class I fumarases of *E*. *coli* (**Table 1**) and *B*. *xenovorans* [10], these data strongly suggest that most, if not all iron-dependent fumarases, are capable to catalyze mesaconate hydration.

## Discussion

This study shows that classical fumarases A and B of *E*. *coli* possess mesaconase activity, and that many (opportunistic) pathogenic *Enterobacteriaceae* have an additional class I fumarase homologue adapted for catalyzing mesaconase reaction. Although the function of this mesaconase in enterobacteria is not known, this enzyme is present in (opportunistic) pathogens and may therefore be related to pathogenicity. Thus, the transcription of the glutamate fermentation genes in *E*. *coli* ATCC 700728 could be dependent on one of the (unknown) stimuli of pathogenicity. Even if so, the further citramalate metabolism in this species is unclear. We cannot exclude the possibility that (*S*)-citramalate is not further metabolized but secreted by pathogens. Interestingly, citramalate, probably of bacterial origin, was detected in plasma from trauma patients [57]. In fact, this compound may inhibit certain enzymes of host metabolism and thus contribute to virulence; for example, citramalate is an inhibitor of mitochondrial aconitase in plants [58]. Altogether, the function of glutamate fermentation cluster in *Enterobacteriaceae* requires further investigations.

Interestingly, the studied *E*. *coli* O157:H7 strain (ATCC 700728) is classified to Biosafety level 2 and is therefore less pathogenic than most other *E*. *coli* O157:H7 strains (Biosafety level 3). Therefore, the strain we chose in our work might be deficient in some pathogenicity-related features, and further studies have to be done either with classical O157:H7 strains or with enterobacteria that have already been shown to express genes involved in the glutamate fermentation [27,56].

Many prokaryotes use the citramalate pathway for isoleucine biosynthesis [59,60,61], and it is possible that citramalate synthesized from glutamate can be used for the formation of isoleucine. However, this process proceeds via the (*R*)-stereoisomer of citramalate [62,63], whereas (*S*)-citramalate in formed in the mesaconase reaction. Therefore, the synthesis of isoleucine from glutamate with participation of FumD would require an epimerase interconverting (*R*)- and (*S*)-citramalate.

Mesaconic, itaconic, methylsuccinic and other C_5_-dicarboxylic acids are regarded as important platform chemicals [64,65,66], and their bioproduction is currently being optimized [65,66,67,68]. The promiscuity of class I fumarases can therefore be important for the optimization of production of these compounds, but also for engineering of (*S*)-citramalate producing strains. Interestingly, *E*. *coli* strain producing up to 7 g/l mesaconate has been engineered recently [66]. It is of particular interest to test the effect of the deletion of class I fumarases on mesaconate production by this strain.

Our bioinformatic analysis identified several putative mesaconases involved in glutamate fermentation through the methylaspartate pathway. The phylogenetic analysis of identified mesaconases and fumarases suggests that mesaconases do not construct a monophyletic cluster on the tree and therefore probably evolved several times from various iron-dependent fumarases during evolution.
